# Sexual Violence Against University Students in Sub-Saharan Africa: A Scoping Review

**DOI:** 10.1177/15248380251320980

**Published:** 2025-02-28

**Authors:** Ester Steven Mzilangwe, Elena González-Rojo, Marie Lindkvist, Isabel Goicolea, Sylvia Kaaya, Faustine Kyungu Nkulu Kalengayi

**Affiliations:** 1 Muhimbili University of Health and Allied Sciences, Tanzania; 2 Umea University, Sweden; 3 University of Lleida, Spain

**Keywords:** sexual violence, sexual harassment, sexual assault, college/university, prevalence, university students, sub-Saharan Africa

## Abstract

Sexual violence (SV) is pervasive on university campuses worldwide, with alarmingly high reported rates in sub-Saharan Africa (SSA). Despite the high reported rates of SV victimization on campuses, existing evidence has not been critically synthesized to give a comprehensive picture of the problem’s extent, common forms, risk factors, and (knowledge about) victims and perpetrators in SSA countries. We aimed to map the existing literature on SV prevalence, types, risk factors, victims, perpetrators, and consequences among university students on campuses in SSA. We included articles focusing on university students from SSA countries published in English or French language from 2014 to 2023. We identified 543 records from nine databases; *Academic Search Premier; CINAHL; EMBASE; MEDLINE; PsychINFO; PubMed; Scopus; SocINDEX*; and *Web of Science*, 82 of which met our inclusion criteria. Retrieved articles covered only one-third of the sub-Saharan region, mostly focusing on female students and victims. Prevalence of different forms of SV varied among countries; authors attributed these variations to differences in time frame, forms of SV, how they were defined, and the tools used. Young female students were identified as common victims, while male teachers and students were common perpetrators. We used the socio-ecological model to summarize risk and protective factors associated with SV victimization and listed the health, social, and economic consequences of SV victimization. Despite these consequences, victims rarely seek help, whether from informal sources or authorities. We call for comprehensive studies in SSA that include both genders and focusing on victims and perpetrators, and address service access barriers.

## Introduction

Sexual violence (SV) is a widespread global public health issue and a violation of sexual rights that affects millions of individuals worldwide, with a disproportionate impact on women (Krug, 2002). SV refers to any sexual act, attempts to obtain a sexual act, unwanted sexual comments or advances, or acts to traffic, or otherwise directed, against a person’s sexuality using coercion, by any person regardless of their relationship to the victim, in any setting, including but not limited to home and work (Krug, 2002). It is particularly prevalent on university campuses, where there are alarmingly frequent reports of high rates (Halstead et al., 2017). Globally, the prevalence of sexual assaults on students in higher education institutions is estimated at 17.5% for women and 7.8% of men (Steele et al., 2023). Compared to other WHO regions, Africa reports the highest prevalence (25.9%) of sexual assault against females in higher education institutions (Steele et al., 2023).

Several factors increase the risk of SV victimization in university settings. Research indicates that being female, young, consuming alcohol and drugs, the year of study, and living off-campus are associated with a higher likelihood of victimization (Beyene et al., 2019; Kefale et al., 2021). Power imbalances, particularly gender inequalities, are fundamental to the perpetuation of SV. For instance, young women may engage in sexual relations with a tutor/lecturer or someone they rely on for support to pass a course necessary for continued financial assistance. This dynamic reinforces women’s subordination and dependency on men and perpetuates violence (Dranzoa, 2018; C. E. Kaufman & Stavrou, 2004). At the institutional level, inadequate monitoring systems, an absence of clear guidelines, and insufficient infrastructure, such as hostels, contribute to the rising victimization of students by SV (Dranzoa, 2018).

To address SV in universities, these institutions have implemented several initiatives, including policies and guidelines regarding prevention and response as well as providing accessible, trauma-informed services (Halstead et al., 2017; Stoner & Cramer, 2019). However, these initiatives face several challenges, including a lack of knowledge about available resources on campus and the unintended consequences of mandatory reporting, like secondary victimization and stigma (Halstead et al., 2017; Munro-Kramer et al., 2024; Perkins & Warner, 2017; Stoner & Cramer, 2019).

Despite the reported high rates of SV victimization on campuses in sub-Saharan Africa (SSA) countries, there is currently no synthesized evidence to provide an overall picture and a clear understanding of not only the extent of the problem but also existing knowledge on potential victims, perpetrators, and consequences of SV victimization in universities. In response to this gap, this scoping review mapped existing literature on SV prevalence, types, risk and protective factors, common victims and perpetrators, consequences, and victims’ response to SV against students on university campuses in SSA and identified evidence gaps.

## Methods

The review adhered to JBI guidelines for scoping reviews (Peters et al., 2015), utilizing the framework proposed by Arksey and O’Malley (2005) and subsequently refined by Levac et al. (2010). We used the Preferred Reporting Items for Systematic Reviews and Meta-analyses extension for Scoping Reviews (PRISMA-ScR) checklist for reporting (Tricco, 1967). The complete checklist is available in the Supplemental Material Table 1. We have registered the protocol of this review with the Open Science Framework and a peer-reviewed journal has published it (Mzilangwe et al., 2024).Information Sources and Search Strategy

Two reviewers (ESM and EGR) independently searched nine databases: *APA PsycINFO; Academic Search Premier; CINAHL; EMBASE; Medline; PubMed; Scopus; SocINDEX*; and *Web of Science*. They performed the search in two rounds, conducting the first round from July 1, 2023 to January 31, 2024 and the second round, which was a supplementary search, from May 2024 to June 2024. Our published protocol outlines the details of the step-by-step search of the database (Mzilangwe et al., 2024). We have provided additional details about the example search process and its results in the Supplemental Material Table 2.

### Study Selection

ESM and EGR assessed the relevance of the retrieved articles based on the inclusion criteria, initially by reviewing the titles and abstracts, followed by a full-text review. They compiled a list of studies that met the criteria. We created and incorporated a detailed flowchart outlining all included and excluded studies, along with the reasons for exclusion into our report. There were no disagreements between the reviewers.

### Eligibility Criteria

We used the population, concepts, and context (PCC) approach to formulate our inclusion criteria (Peters et al., 2015). Articles were eligible if they focused on: (a) human subjects who are university students of both sexes, aged 18 years and above (Population); (b) SV on campuses (Concept); and (c) universities in SSA (Context) from the inception until December 31, 2023. We excluded articles with studies conducted in populations other than students, settings other than universities, areas other than SSA, without focus on SV in relation to the concepts listed above, and those published in a language other than English or French. Due to the large number of articles retrieved, we narrowed the aim of this article to the first specific objective of the protocol (Mzilangwe et al., 2024), and we excluded articles published before 2014 in all sections of the results except for the sources geographical coverage map.

### Charting, Summarizing and Reporting the Results

We used the Excel program to prepare the data charting form. The form included the name of the first author, year of publication, study location (country), aims of the study, study design, study population, sample size, SV terminology used, measurement tools, time frame, and the findings. We used a broader narrative review to analyze information based on the context of the study findings and their conclusion. Based on overlaps and diversities of the findings, we developed a thematic framework to present a narrative account of all extracted information from the articles to answer our research questions. We then summarized the extracted data into different themes, including: (a) study characteristics, (b) concepts and definitions used in the literature, (c) theories, models and framework used by authors, (d) prevalence, (e) risk and protective factors, (f) victims and perpetrators, (g) health and social-economic consequences, and (h) victims’ disclosures and reporting of SV victimization. The last five themes focused on the aim, while the first three themes were intentionally included to enhance the reader’s understanding and interpretation of the presented data. We employed tables, figures, and a map to organize and summarize the data.

## Results

**Figure 1.**
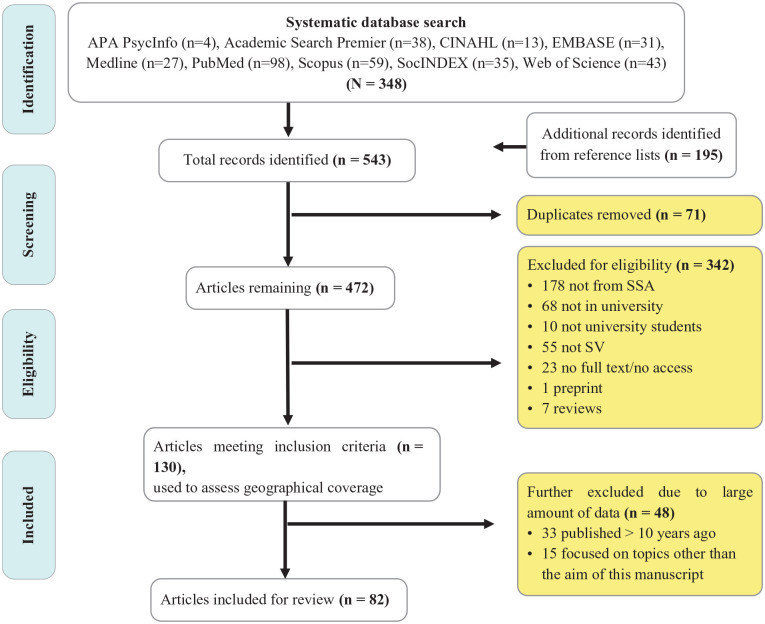
Flow diagram of the identified articles from the searched databases.

Our search retrieved 543 articles, of which we excluded 413 based on various exclusion criteria, leaving 130 articles (see Figure 1). We examined geographical coverage using all 130 articles; however, due to the volume of retrieved articles, we have narrowed our focus in the subsequent sections of this review to 82 articles published in the last 10 years (2014–2023).

### Study Characteristics

**Figure 2.**
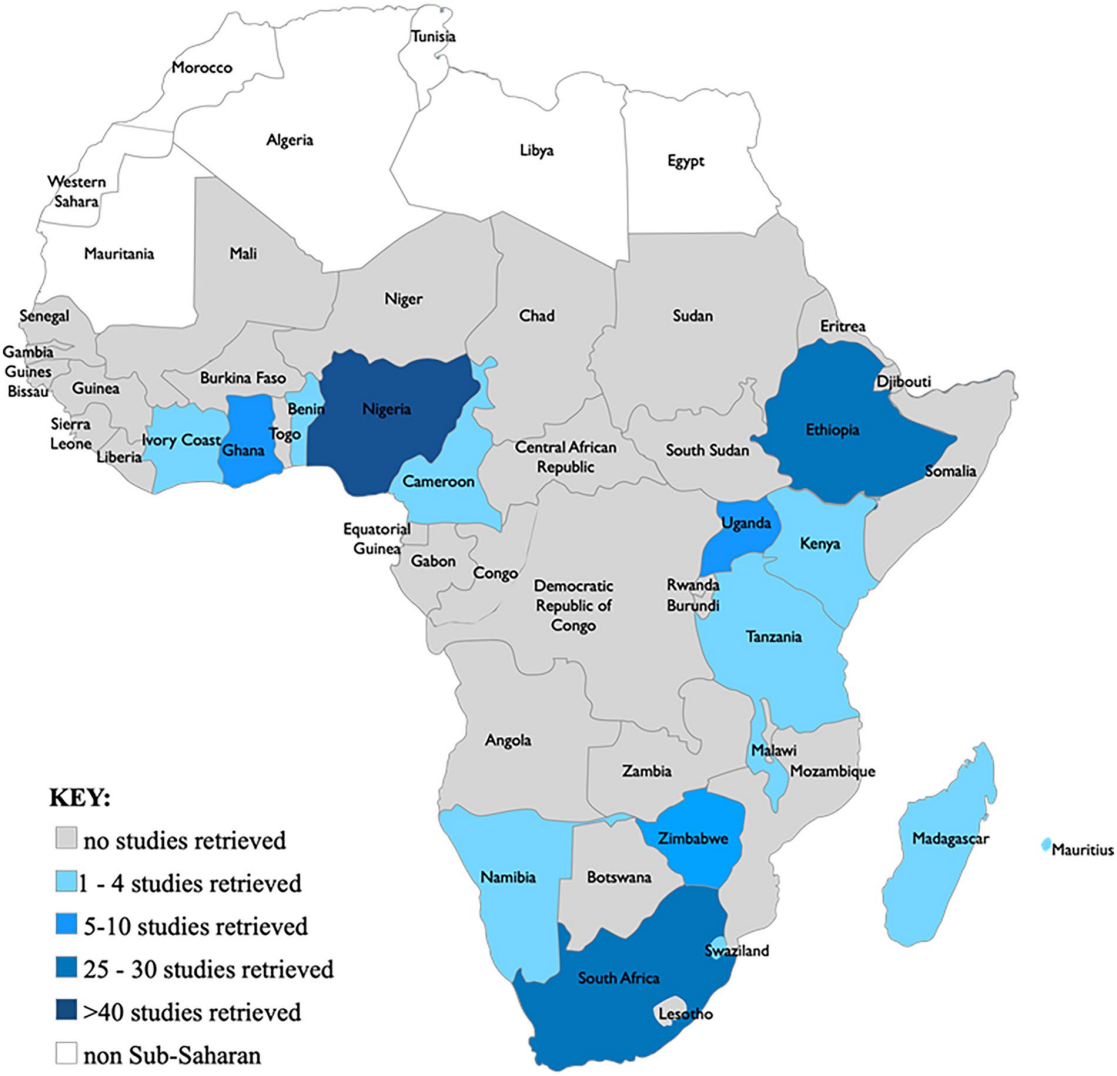
A map of SSA indicating countries with published articles on SV against university students from inception to December 31, 2023. *Note*. SV = sexual violence; SSA = sub-Saharan Africa. *Not included in the definition for some articles.

The articles (*n* = 130) were from 16 countries, covering only one-third of the sub-Saharan region; however, three-quarters of them were from Nigeria (*n* = 43), followed by South Africa (*n* = 29) and Ethiopia (*n* = 27). One article involved seven countries (Cameroon, Ivory Coast, Madagascar, Mauritius, Namibia, Nigeria, and South Africa). Figure 2 provides details of geographical representation.

Among the 82 articles we summarized, 75 (91.5%) utilized primary data, while five used both primary and secondary data. The remaining two articles utilized only secondary data. Articles used both qualitative and quantitative methods, with 38 articles using quantitative while 32 used qualitative and 12 utilized mixed methods. Female students were the targeted population in half (*n* = 42) of the articles, with three focusing on only male populations, while the rest included both male and female students (*n* = 34). The topics addressed consisted of various areas, including prevalence, risk and protective factors, perpetration, victims’ experiences and coping mechanisms, knowledge, perceptions, myth, consequences, help-seeking and reporting of SV victimization against students in universities.

### Concepts and Definitions Used in the Literature

**Figure 3.**
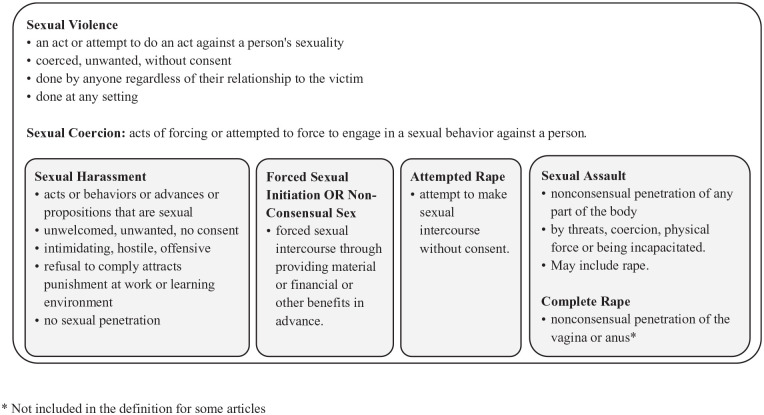
Constructed definition of sexual violence based on retrieved articles.

The authors used different terminologies to describe different forms of SV; some authors used more than one terminology. The most typical term used was “sexual violence”; others include “dating violence,” “sexual coercion,” “sexual harassment,” “forced sexual initiation,” “non-consensual sex,” “sexual assault,” “rape,” “attempted rape,” “completed rape,” “date rape,” and “coercive sexual practices.” Most authors included the definitions of specific forms of SV terminologies used in their research, citing World Health Organization documents and previous published articles. We analyzed the definitions that authors provided by extracting the defining elements and comparing them for similarities and differences. Figure 3 represents a construction of how the retrieved articles defined various forms of SV.

The definition of the term “sexual violence” was broad as it included any behavior or act performed against one’s sexuality and against one’s will. Sexual harassment definition included elements of forms of behaviors, the intention of such behaviors, lack of consent, the outcome of such behaviors and the environment where such behaviors occur and excluded statements that imply forceful sexual penetration. Definitions of rape narrowed down to acts of sexual penetration (by a penis, a body part, or an object), use of force, and the lack of consent. Only two articles out of nine included anal penetration when defining rape (Abubeker et al., 2021; Abulie & Tesfaye, 2014). Further divisions of rape were date rape, attempted rape, and completed (or performed) rape. Date rape implies conducts of such behavior by a dating partner. The definition of attempted rape was “an attempt” or “a trial” to commit an act of rape and complete rape as successful penetration. The definition of sexual assault contained defining elements that were similar to completed rape; however, one article suggested that the use of the terms “sexual assault” and “sexual harassment” can be interchangeable (Rebecca et al., 2019).

### Theories, Models, and Framework Used by Authors

Few articles (*n* = 17) explicitly described the theories or frameworks that they utilized in their analysis. We have summarized them into three groups: those focusing on organizational and learning perspectives; gender perspectives; and others. The organizational and learning group of theories include organizational theory, social exchange theory, the socio-ecological model theory, transformative learning theory, the confluence model, socio-cultural theory, social learning theory, differential association, crime theory gap in knowledge theory, and Bourdieu’s theory of practice (Adams et al., 2016; Bezabeh, 2016; Compton et al., 2023; Effiom et al., 2019; Gbagbo et al., 2023; Kebirungi, 2021; Manik & Tarisayi, 2021; Okechi, 2023; Olaigbe & Fagbenro, 2021; Y. L. Olaleye & Ezeokoli, 2016; Treffry-Goatley et al., 2018; Umana et al., 2014). Only five articles used gender-centered theories in their research; these include theories of intersectionality, post-structural conceptualizations of power, hegemonic masculinity theory, betrayal trauma theory, tripartite model of sexual harassment, and male dominance theory (Anderson & Naidu, 2022; Okafor et al., 2022; Sidelil et al., 2022; Singh, 2023). Others include natural/biological theories and structuration/collective conscience models (Adams et al., 2016; Kanyemba & Naidu, 2022).

### Prevalence of Reporting Different Forms of SV in Different Time Frames

Over a third of the articles (*n* = 31) report the prevalence of different forms of SV across 11 countries: Ethiopia (*n* = 16), Nigeria (*n* = 7), South Africa (*n* = 5), Ghana (*n* = 1), Tanzania (*n* = 1), and one article included data from Nigeria, South Africa, and five additional countries (Cameroon, Ivory Coast, Madagascar, Mauritius, and Namibia). Half of those articles (*n* = 16) reported the prevalence of one form of SV, while the other half (*n* = 15) reported more than one form of SV. The articles assessed prevalence over different time frames: lifetime; post-university enrolment; and in the past 12 months. For more detailed information, please refer to Supplemental Material Table 3.

**Figure 4.**
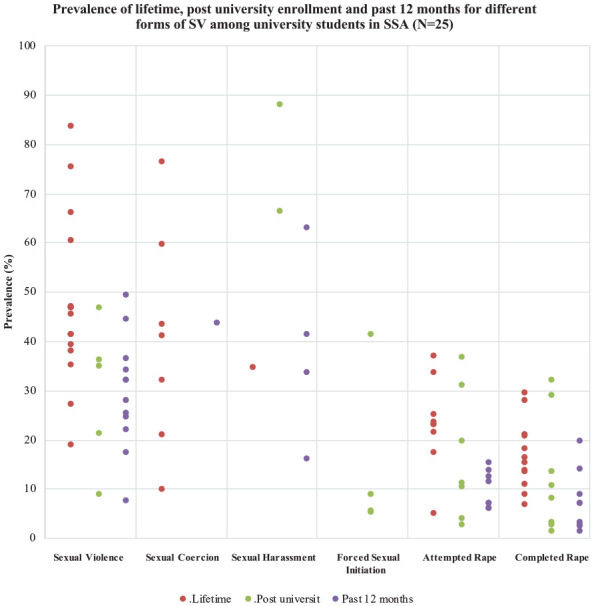
Prevalence of different forms of SV against university students at different time frame from six countries (Ethiopia, Eswatini, Ghana, Nigeria, South Africa, and Tanzania). *Note*. SV = sexual violence.

We selected and summarized prevalence data using dot plots (Figure 4) from 25 articles that reported on various forms of SV, including sexual coercion, harassment, forced initiation, attempted rape, and completed rape (Abubeker et al., 2021; Abulie & Tesfaye, 2014; Ajayi et al., 2021; Andualem et al., 2016; Benti & Teferi, 2015; Birkie et al., 2020; Esayas et al., 2023; Gebrie et al., 2022; Kassa et al., 2019; Margaret et al., 2014; Mosha et al., 2022; Mutinta, 2022; Negero et al., 2019; Odufuye & Ajuwon, 2020; Ogunfowokan et al., 2023; Oni et al., 2019; Prosper et al., 2014; Rebecca et al., 2019; Sarah, Cheryl, et al., 2017; Temesgan et al., 2021; Tolesa et al., 2015; Tolesa et al., 2014; Workye et al., 2023; Yemsrach Kebede et al., 2017; Yohannes Mehretie & Mihiret Abreham, 2017). Furthermore, we excluded data from six articles focusing on other forms of SV and those without specified time frames (Akpunne et al., 2020; Almaz et al., 2015; Boladale et al., 2015; M. T. Machisa et al., 2021; Pengpid & Peltzer, 2016; Umana et al., 2014).

The summarized data revealed that lifetime SV victimization was slightly more prevalent compared to post-university enrolment and in the past 12 months. Moreover, instances of SV, coercion, and harassment were more prevalent compared to forced sexual initiation, attempted rape, and completed rape. Sexual harassment had the highest prevalence after enrolment and in the past 12 months, with a prevalence of 88% (Tanzania) and 63% (Ethiopia), respectively. Ethiopia had the highest prevalence of attempted and completed rape after university enrolment, with 37% and 32%, respectively, while incidents in the past 12 months were 15% (Ethiopia) and 20% (Eswatini), respectively.

### Types of Research Instruments Used to Measure the Prevalence of SV

The tools used to measure the prevalence of SV victimization varied in the number and type of items and the guidance provided to participants. Most tools included specific questions or statements, while a few allowed participants to share their perspectives more freely. Table 1 presents the summary of the names of tools and type of SV measured. The most widely used questionnaire used was the WHO Multi-Country Study on Women’s Health and Domestic Violence against Women Questionnaire. Other tools were the Sexual Experiences Survey–Short Form Version (SES-SFV), the Conflict Tactic Scale–Revised (CTS-R), and the Sexual Abuse History Questionnaire. Twelve articles developed their tools using literature from previous studies, and five did not provide information on the tools they used in their studies. More detailed information is available in Supplemental Material Table 3.

**Table 1.** Different (Types of) Research Instruments Used to Measure the Prevalence of SV.

Instrument	Types of Sexual Violence measured	Authors (Year)
Conflict Tactic Scale–Revised (CTS-R)	Sexual Violence	M. Boladale et al. (2015), S. Pengpid and K. Peltzer (2016)
Sexual Experiences Survey–Short Form Version (SES-SFV)	Sexual Violence, Sexual Harassment, Attempted Rape, Completed Rape, Sexual Coercion and Sexual Assault	A. A. Ogunfowokan et al. (2023), B. C. Akpunne et al. (2020), Rebecca et al. (2019)
Sexual Abuse History Questionnaire	Sexual Violence	M. Birkie et al. (2020)
WHO Multi-Country Study on Women’s Health and Domestic Violence against Women Questionnaire	Sexual Violence, Sexual Harassment, Attempted Rape, Completed Rape and Sexual Coercion	Andualem et al. (2016), B. Tolesa et al. (2015), H. L. Esayas et al. (2023), J. E. Umana et al. (2014), M. G. Negero et al. (2019), M. T. Machisa et al. (2021), Tolesa et al. (2014), Yohannes Mehretie & Mihiret Abreham (2017)

### Multifaceted levels of risk and protective factors associated with SV victimization among university students

**Figure 5.**
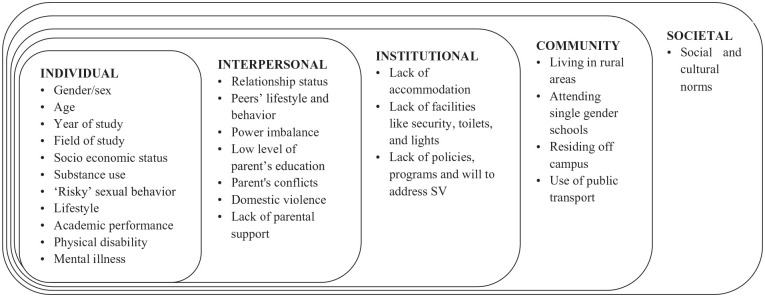
Risk factors for sexual violence victimization grouped according to the socio-ecological model.

We used the socio-ecological model to describe and group the risk factors reported in the articles. The socio-ecological model is a theoretical framework used to understand the complex interplay between individual (biological and personal), interpersonal (social relationships), organizational/institutional (e.g., university), community (settings such as residential areas and neighborhoods where social relationships occur), and societal factors (broad social and cultural norms) that influence human behavior and health outcomes (Kilanowski, 2017). It provides a comprehensive approach to understanding and addressing the multiple levels of factors that influence health behaviors and outcomes. The socio-ecological model was specifically applied to highlight the complexity and various levels of risk and protective factors contributing to victimization or perpetration of SV (Figure 5). We provide a summary below, and more detailed information in Supplemental material Table 4.

#### Individual Level

Articles reported association of individual factors, such as sex, age, year of study, poor economic status, substance use (alcohol, khat chewing, and cigarette smoking), with SV victimization. Participant narratives from qualitative studies elaborate that they perceived female students as common victims of SV compared to male students; however, results from quantitative studies indicate that male students were also victims, although to a lesser extent compared to their female counterparts. In addition, there was a perception that young students in their early years at university were less experienced with university life and less knowledgeable of where to get needed services and therefore more likely to be a target for SV (R. A. Aborisade, 2014; Abubeker et al., 2021; Abulie & Tesfaye, 2014; Ajayi et al., 2021; Akpunne et al., 2020; Anderson & Naidu, 2022; Andualem et al., 2016; Bello, 2020; Benti & Teferi, 2015; Birkie et al., 2020; Boladale et al., 2015; Esayas et al., 2023; Gukurume & Shoko, 2023; Kanyemba & Naidu, 2019; M. R. Kaufman et al., 2019; Mafa & Simango, 2021; Margaret et al., 2014; Mehra et al., 2014; Mutinta, 2022; Negero et al., 2019; Odufuye & Ajuwon, 2020; Oni et al., 2019; Peace et al., 2015; Pengpid & Peltzer, 2016; Sadati & Mitchell, 2021; Sarah, Cheryl, et al., 2017; Sikweyiya et al., 2023; Temesgan et al., 2021; Tolesa et al., 2014; Umana et al., 2014; Workye et al., 2023; Yemsrach Kebede et al., 2017; Yohannes Mehretie & Mihiret Abreham, 2017). Qualitative study findings also showed that sometimes men would get the female students drunk or drug them prior to sexually abusing them (Anderson & Naidu, 2022; Dumbili & Williams, 2020; Margaret et al., 2014; Singh, 2023; Treffry-Goatley et al., 2018). Notably, many of the articles that reported on associations between risk/protective factors and SV did not include males as potential victims, which made it impossible to establish comparisons. Moreover, in other settings, there were conflicting results whereby students of older age and those with monthly incomes compared to those with no income were more likely to report experiencing SV (Gebrie et al., 2022; Mutinta, 2022; Sarah, Cheryl, et al., 2017), while the same were reported as a protective factors in another quantitative study (Kassa et al., 2019).

Students with poor academic performance, physical disability, and mental health challenges such as having depression and PTSD symptoms and neuroticism personality were more likely to be victims of SV. Qualitative findings further elaborate that female students who were perceived as having weak academic performance were more likely to be targeted by those in power, including academicians (Benti & Teferi, 2015; Boladale et al., 2015; Effiom et al., 2019; Eller, 2016; Gbagbo et al., 2023; M. R. Kaufman et al., 2019; Negero et al., 2019; Olaigbe & Fagbenro, 2021; Pengpid & Peltzer, 2016; Sikweyiya et al., 2023).

#### Interpersonal Level

Authors reported that the lifestyle and behavior of peers, friends, or partners influence the likelihood of experiencing SV; these included drinking on a regular basis or having a first partner who was more than 4 years older (Andualem et al., 2016; Gebrie et al., 2022; Sarah, Cheryl, et al., 2017; Yohannes Mehretie & Mihiret Abreham, 2017). Qualitative studies suggested that men were using violence to show their power; for example, lecturers were using their power and influence to harass female students, especially those they perceived as weak academically (Benti & Teferi, 2015; Eller, 2016; Gbagbo et al., 2023; M. R. Kaufman et al., 2019; Kebirungi, 2021; Olaigbe & Fagbenro, 2021; Sikweyiya et al., 2023; Yunusa & Usman, 2023). In one study, participants from a focused group discussion said that saying no to a professor or reporting him was dangerous, and that students avoided bruising the professor’s ego (Eller, 2016).

Parental conditions like witnessing interparental conflicts, coming from tight family control, and not discussing personal issues with family were also among the risk factors to SV victimization, while protective factors included having a literate father, receiving financial support from the family and being female college students who have family discussions on reproductive health and related personal issues with their parents (Ajayi et al., 2021; Birkie et al., 2020; Gebrie et al., 2022; Kassa et al., 2019; Negero et al., 2019; Temesgan et al., 2021; Tolesa et al., 2015; Tolesa et al., 2014; Umana et al., 2014; Yohannes Mehretie & Mihiret Abreham, 2017).

#### Institutional Level

A lack of essential facilities for security, such as fences around dormitories, lights in certain areas on campus, shortages of toilets and accommodation for female students, and the availability of substances of abuse on campus, increases the likelihood of SV. In addition, a lack of institutional willingness to introduce policies and programs to address SV matters in universities, a lack of knowledge, awareness and willingness to report SV, and the negative attitudes of university staff and management toward preventing SV were reported to contribute to a high prevalence of SV on campuses. In addition, poor adherence to professional ethics was identified as one of the moral dilemmas that led to instructors engaging in sexual relationships with students (Aina-Pelemo et al., 2021; Anderson & Naidu, 2022; Andualem et al., 2016; Bello, 2020; Eller, 2016; Gbagbo et al., 2023; Kebirungi, 2021; Manik & Tarisayi, 2021; Negero et al., 2019; Okafor et al., 2022; Olaigbe & Fagbenro, 2021; Ross et al., 2022; Sadati & Mitchell, 2021; Sidelil et al., 2022; Sikweyiya et al., 2023; Yunusa & Usman, 2023).

#### Community Level

Students who grew up in a rural area and had attended single-gender schools were more likely to report an experience of SV (Negero et al., 2019; Prosper et al., 2014; Yohannes Mehretie & Mihiret Abreham, 2017). However, in one study, living in rural areas was a protective factor (Tolesa et al., 2014). Residing off campus or more than 20 min away from the campus areas, use of public transport, and overcrowded vehicles increased the likelihood of experiencing SV (Eagle & Kwele, 2021; Mosha et al., 2022; Mutinta, 2022; Opekitan et al., 2019; Pengpid & Peltzer, 2016; Prosper et al., 2014; Temesgan et al., 2021).

#### Societal Level

Traditional gender roles and relationship dynamics, such as the expectation for females to have sex when in a relationship, patriarchal systems, toxic masculinity, gender stereotyping, “rape myth acceptance,” and religious beliefs were elaborated as contributors to SV perpetration by men. In some studies, participants perceived the dressing of female students as “provocative and indecent,” or women as “being desperate” or “being at fault” because “sometimes they say no, when they mean yes,” while men are perceived of having a high libido and unable to control themselves once aroused (R. A. Aborisade, 2016; Babatunde & Falaye, 2022; Bashonga & Khuzwayo, 2017; Bello, 2020; Boateng et al., 2023; Compton et al., 2023; de Villiers et al., 2021; Eller, 2016; Gukurume & Shoko, 2023; Kanyemba & Naidu, 2019; Kanyemba & Naidu, 2022; M. R. Kaufman et al., 2019; Matthews et al., 2018; Michelle et al., 2019; Nafuka & Shino, 2014; Okechi, 2023; Olaigbe & Fagbenro, 2021; Peace et al., 2015; Sadati & Mitchell, 2021; Sarah, Eugene Kofuor Maafo, et al., 2017).

### Common Perpetrators and Victims of SV Within and Outside University Campuses

Perpetrators of SV were predominantly males and those who were known to or had close relationships with the victim, while victims were predominantly females (Abulie & Tesfaye, 2014; Bezabeh, 2016; Gebrie et al., 2022; Kanyemba & Naidu, 2022; Mafa & Simango, 2021; Manik & Tarisayi, 2021; Negero et al., 2019; Rebecca et al., 2019). Male students were also among victims of sexual harassment, however not commonly reported to experience rape (O. S. Olaleye & Ajuwon, 2019; Pengpid & Peltzer, 2016). In addition, studies reported that students living with disability, and members of the LGBTQ community were also frequently victims (Gbagbo et al., 2023; Manik & Tarisayi, 2021). At the university, common perpetrators include boyfriends, close friends, fellow university students, university teachers, university staff, and strangers to the university area. From the family and community, perpetrators included husbands/intimate partners, parents, other family members, friends, neighbors, and strangers. The typical locations for the perpetration of SV include teachers’ or staff offices, classrooms, library, school halls, students’ residences, and on the campus grounds. However, some of the incidences of SV occur outside the campus; these included perpetrators’ homes, hotels, victims’ homes, victim friends’ homes, at party venues, and other places (Abulie & Tesfaye, 2014; Esayas et al., 2023; M. R. Kaufman et al., 2019; Mahabeer, 2021).

### Health and Social-Economic Consequences of SV

**Figure 6.**
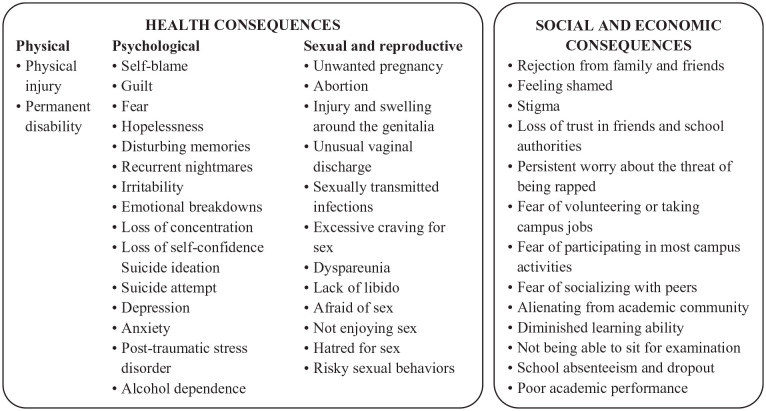
Health and social-economic consequences of SV victimization against students in SSA. *Note*. SV = sexual violence; SSA = sub-Saharan Africa.

SV had detrimental effects on students, not only changing their lives at university but also affecting their academic outcomes. We summarized these effects (Figure 6) into health (physical, psychological, sexual, and reproductive) and social-economic consequences. Detailed information is provided in Table 4 in the Supplemental material.

The reported physical health consequences include physical injury and permanent disability (Negero et al., 2019; Ross et al., 2022; Umana et al., 2014). Psychological effects include self-blame, guilt, fear, hopelessness, disturbing memories, recurrent nightmares, emotional breakdowns, suicide ideation, and attempt. Others include loss of concentration, loss of self-confidence, irritability, depression, anxiety, post-traumatic stress disorder, alcohol dependence, and other mental health related issues (Akintayo Olamide et al., 2017; Almaz et al., 2015; Anderson & Naidu, 2022; Gbagbo et al., 2023; Gebrie et al., 2022; Konlan & Dangah, 2023; M. T. Machisa et al., 2022; Margaret et al., 2014; Negero et al., 2019; Olaigbe & Fagbenro, 2021; Ross et al., 2022; Sadati & Mitchell, 2021; Umana et al., 2014). The sexual and reproductive health consequences varied from unwanted pregnancy some ending into an abortion, injury and swelling around the genitalia, unusual vaginal discharge, sexually transmitted infections, and other sexual dysfunction problems (Abulie & Tesfaye, 2014; Gebrie et al., 2022; Mutinta, 2022; Sadati & Mitchell, 2021; Umana et al., 2014; Workye et al., 2023). SV incidences affect social life of victims in many ways, including rejection from family and friends, feeling ashamed, stigma, loss of trust for friends and school authorities, and being worried about being raped every time they were walking alone at night, which affects their social and academic performance. Moreover, victims experienced academic-related adverse outcomes like diminished learning ability, not being able to sit for examination after rape, school absenteeism and dropout, and low school performance (Anderson & Naidu, 2022; Eller, 2016; Gbagbo et al., 2023; Gebrie et al., 2022; Mafa & Simango, 2021; Mafa et al., 2022; Melak & Singh, 2021; Mutinta, 2022; Negero et al., 2019; Olaigbe & Fagbenro, 2021; Ross et al., 2022; Sadati & Mitchell, 2021; Shakila et al., 2015; Umana et al., 2014; Workye et al., 2023).

### Victims’ Disclosure and Low Reporting Rates of Incidences of SV

**Figure 7.**
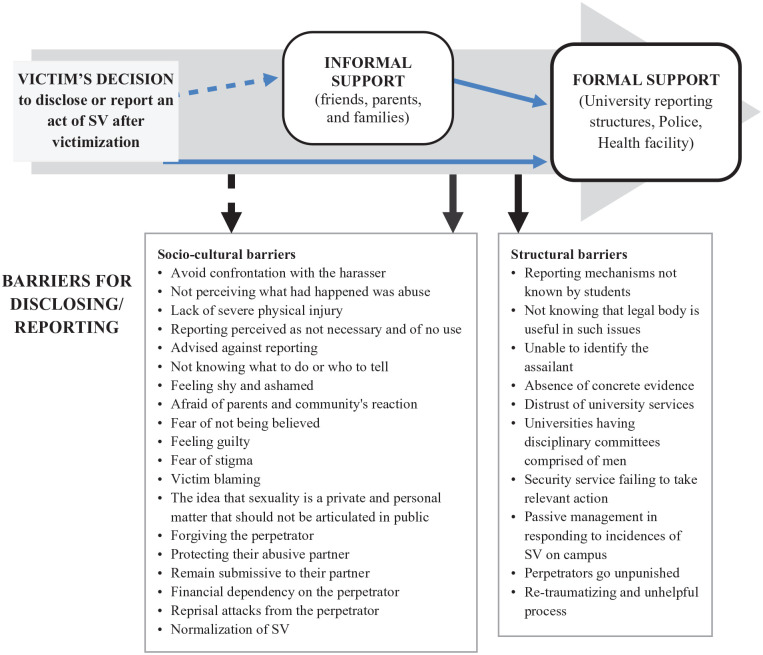
Socio cultural and structural barriers of disclosure and reporting incidences of SV victimization against students in SSA. *Note*. SV = sexual violence; SSA = sub-Saharan Africa.

Most articles reviewed indicated low rates of help-seeking (0%–46%) by most victims of SV. In the few cases when SV victims seek help, they prefer to use informal support from friends, parents, and families (R. A. Aborisade, 2014; Abulie & Tesfaye, 2014; Benti & Teferi, 2015; M. R. Kaufman et al., 2019; Odufuye & Ajuwon, 2020; Y. L. Olaleye & Ezeokoli, 2016; Yohannes Mehretie & Mihiret Abreham, 2017). Although some universities have established formal response services, the rate of reporting SV victimization varied between 0% and 8.1%. Likewise, the reporting rate to other formal authorities, like the police, ranged from 4.7% to 8.4% (Abulie & Tesfaye, 2014; Adams et al., 2016; Benti & Teferi, 2015; Bezabeh, 2016; Mapayi et al., 2023; Ogunfowokan et al., 2023; Yohannes Mehretie & Mihiret Abreham, 2017). We summarized the various reasons for not reporting incidences of SV into socio-cultural and structural reasons in Figure 7. The socio-cultural barriers prevented victims from reporting to both formal and informal support systems. In contrast, structural barriers primarily impacted the reporting to formal support systems.

## Discussion

This scoping review synthesized 82 articles and provided an overall picture of SV against university students in SSA. Most studies coming from just three countries and covering only one-third of the region. There are inconsistencies in concepts and terminologies, and few articles reference theoretical frameworks. Various tools measured different types of SV over different time frames, leading to varied prevalence rates. Risk and protective factors for SV were identified at multiple levels. Most victims were female students, while perpetrators were predominantly male partners. The findings highlight both health and socio-economic consequences of SV. Reporting, disclosing, and seeking help for SV are uncommon due to cultural and structural barriers.

Despite proportionally higher rates of SV in SSA when compared to other regions, and a growing interest in the field since 2010, the retrieved articles came from only 16 of the 49 SSA countries, and in particular from three of those. This geographical disparity in publications counts as one of the critical research gaps captured from this review. Most studies were observational, did not integrate a theoretical framework, and focused on female students and victims. The retrieved articles categorized gender within the traditional binary of male and female, resulting in a lack of data on the experiences of LGBTQIA+ students.

The reported prevalence of various forms of SV varied among countries and ranged from 1% (past 12 months experience of complete rape) to 88% (experience of sexual harassment after joining university). However, there were huge variations in the prevalence data, which made it difficult to make sense of commonalities among studies and across countries. The differences in the setting and country-specific contextual factors could be an explanation for these variations. Moreover, the forms of SV assessed the [different] definitions of the terminologies and differences in the time frames, and measurement tools used may also contribute to these variations. From this review, we observed the use of 11 different terminologies to refer to different types of SV. Despite most authors describing the various forms of SV with standard definitions, several articles lacked definitions while a few excluded important elements in their definition; for example, several authors excluded “anal” and “oral” penetration when defining rape. This gap may have affected how authors measured and interpreted different acts of SV and make comparison difficult.

Moreover, we observe less use of theories and frameworks in most of the articles and even, when used, few studies take a gender perspective. This may have affected how researchers formulated and interpreted the findings of their studies, and they were hence less likely to address complex gender-related elements intertwined in SV victimization.

Variations of instruments that measure SV have been a global challenge, frequently reported in other reviews as one of the identified research gaps (Basile et al., 2014; Fedina et al., 2018; Halstead et al., 2017; Stoner & Cramer, 2019). We noted that the majority of studies utilized the WHO Multi-Country Study on Women’s Health and Domestic Violence against Women Questionnaire, which focuses primarily on intimate partner violence, potentially overlooking other forms of violence and abuse. Despite efforts to develop standardized elements to measure campus SV (Basile et al., 2014), the question remains whether defining and measuring SV can accommodate cultural contexts and regional variations.

There are several multifaceted levels of complexity in the risk and protective factors associated with SV victimization among university students, the majority of victims being female students, while commonly reported perpetrators are male peers and teachers. However, to a lesser extent, male students were also at risk, and this was a similar finding from other reviews (Bhochhibhoya et al., 2021; Klein & Martin, 2021; Steele et al., 2023; Tashkandi et al., 2023). Quantitative studies highlight the association between various individual characteristics of victims and their experience of SV; however, only one study focused on perpetrators. A focus on analyzing risk factors for SV victimization can potentially suggest victim-blaming by shifting the focus to the victims’ characteristics rather than the characteristics of the perpetrators. This can further misinform interventions whose design is most likely targeting victim characteristics as the reasons for violence, leaving the role of addressing SV in the hands of the victim rather than addressing perpetrators’ characteristics and behavior as well as structural issues as more likely determinants of the occurrence of SV.

The findings show that SV victimization results in adverse physical, psychological, reproductive health and social consequences. However, despite these effects, we found consistently low rates of disclosure and formal service utilization among students suggesting barriers to disclosure and access to available services that allow the perpetuation of SV.

### Limitations

Although this review focused on the sub-Sahara region, the reviewers were limited to two languages, English and French, and all retrieved articles were published in English. The search terms and the inclusion criteria used in this study may have limited the results. However, given the broad aims of this review, we analyzed vast thematic areas and generated comprehensive evidence on SV victimization against university students in SSA.

## Conclusion

This scoping review reveals that, despite increasing interest in research on SV, we were able to retrieve articles from only one-third of all countries in SSA. This suggests a scarcity of research on SV victimization among university students and significant gaps in the geographical coverage of SV research in this region. Most studies were observational, focusing on female students as victims and overlooking male student victims, with fewer studies focusing on perpetrators. Compared to other regions, SV victimization against university students is higher in SSA. Moreover, we found an alarming scarcity of studies analyzing SV from a gender perspective, inconsistencies in defining and measuring SV, and a focus on risk factors that can lead to re-victimization while missing the links with gender-unequal relations and power, which risks providing a simplistic picture of SV. The results highlight various factors associated with experiencing SV layered at different socio-ecological levels that may require multilevel interventions and strategies. Despite the reported adverse outcomes of SV for affected students, very few seek help from either informal supports or relevant authorities, emphasizing the need to improve access to support services. We call for more comprehensive studies in SSA that focus on both male and female students, including both victims and perpetrators. These studies should also address barriers to accessing services and support. Additionally, it is crucial to identify and mitigate risk factors while reinforcing protective factors. The use of theoretical frameworks is essential to guide these studies, ensuring a robust and systematic approach to understanding and addressing the issues. Researchers, governments, institutions, non-government organizations, and other stakeholders need to collaborate in addressing these gaps and ensure the development and implementation of effective prevention and support systems to tackle the problem of SV in SSA universities.

**Table:** Summary of Critical Findings.

1. Despite higher SV rates and growing interest since 2010, research articles were found from only one-third of SSA countries, indicating significant gaps. 2. SV victimization among university students is higher in SSA compared to other regions like North America and Europe, with prevalence varying significantly due to setting, contextual factors, definitions, time frames, and measurement tools. 3. Most studies lacked theoretical frameworks, had inconsistencies in definitions used for SV, primarily focused on female students as victims, and less on perpetrators. 4. Factors associated with SV are complex and exist at various socio-ecological levels, indicating the need for multilevel interventions. Moreover, focusing solely on individual risk factors can lead to re-victimization and overlook gender-unequal relations and power dynamics. 5. Despite the adverse outcomes of SV, very few student seek help from either informal supports or authorities, emphasizing the need to improve access to support services.

**Table:** Implications for Practice, Policy and Research.

1. We call for more studies (on SV) across SSA to address geographical gaps. 2. Standardized measurement tools and broader definitions are needed for reliable SV assessment and to allow comparison. 3. Longitudinal research is essential to establish causal relationships and understand the long-term effects of SV. 4. Studies should incorporate gender related theories when researching SV to better understand the structural factors that increase vulnerability and perpetration. 5. We call for more research on how sexual orientation, disability, and ethnicity affect vulnerability to SV. Additionally, studying potential perpetrators is crucial to understand SV mechanisms and develop effective, non-stereotypical prevention strategies. 6. Universities should establish clear policies, enhance access to culturally and gender-sensitive support services, create peer support programs, launch awareness campaigns, and regularly assess initiatives to foster a supportive environment where students feel comfortable seeking help and using formal services.

## Supplemental Material

sj-docx-1-tva-10.1177_15248380251320980 – Supplemental material for Sexual Violence Against University Students in Sub-Saharan Africa: A Scoping Review Supplemental material, sj-docx-1-tva-10.1177_15248380251320980 for Sexual Violence Against University Students in Sub-Saharan Africa: A Scoping Review by Ester Steven Mzilangwe, Elena González-Rojo, Marie Lindkvist, Isabel Goicolea, Sylvia Kaaya and Faustine Kyungu Nkulu Kalengayi in Trauma, Violence, & Abuse
