# Supplementary material for: Sexual Violence Against University Students in Sub-Saharan Africa: A Scoping Review
Source: Trauma Violence Abuse. 2025 Feb 28;27(3):542–57. doi: 10.1177/15248380251320980 (PMC13287379; doi:10.1177/15248380251320980)
Supplement: sj-docx-1-tva-10.1177_15248380251320980 – Supplemental material for Sexual Violence Against University Students in Sub-Saharan Africa: A Scoping Review [file sj-docx-1-tva-10.1177_15248380251320980.docx]

# APPENDIX

## Table 1: Preferred Reporting Items for Systematic reviews and Meta-Analyses extension for Scoping Reviews (PRISMA-ScR) Checklist^[[1]](#footnote-1)^

| **SECTION** | **ITEM** | **PRISMA-ScR CHECKLIST ITEM** | **PAGE #** |
| --- | --- | --- | --- |
| **TITLE** | | | |
| Title | 1 | Identify the report as a scoping review. | pg 1; line #1 |
| ABSTRACT | | | |
| Structured summary | 2 | Provide a structured summary that includes (as applicable): background, objectives, eligibility criteria, sources of evidence, charting methods, results, and conclusions that relate to the review questions and objectives. | pg 1; line #2-22 |
| **INTRODUCTION** | | | |
| Rationale | 3 | Describe the rationale for the review in the context of what is already known. Explain why the review questions/objectives lend themselves to a scoping review approach. | pg 2; line #26-p2 3; line #59 |
| Objectives | 4 | Provide an explicit statement of the questions and objectives being addressed with reference to their key elements (e.g., population or participants, concepts, and context) or other relevant key elements used to conceptualize the review questions and/or objectives. | pg 3; line #59-62 |
| **METHODS** | | | |
| Protocol and registration | 5 | Indicate whether a review protocol exists; state if and where it can be accessed (e.g., a Web address); and if available, provide registration information, including the registration number. | https://bmjopen.bmj.com/content/bmjopen/14/1/ e076015.full.pdf |
| Eligibility criteria | 6 | Specify characteristics of the sources of evidence used as eligibility criteria (e.g., years considered, language, and publication status) and provide a rationale. | pg 4; line #89-99 |
| Information sources | 7 | Describe all information sources in the search (e.g., databases with dates of coverage and contact with authors to identify additional sources), as well as the most recent search was executed. | pg 3; line #73-76 |
| Search | 8 | Present the full electronic search strategy for at least 1 database, including any limits used, such that it could be repeated. | pg 4; line #79-80, supplementary materials Table #2 |
| Selection of sources of evidence† | 9 | State the process for selecting sources of evidence (i.e., screening and eligibility) included in the scoping review. | pg 4; line #76-87 |
| Data charting process‡ | 10 | Describe the methods of charting data from the included sources of evidence (e.g., calibrated forms or forms that have been tested by the team before their use, and whether data charting was done independently or in duplicate) and any processes for obtaining and confirming data from investigators. | pg 5; line #102 |
| Data items | 11 | List and define all variables for which data were sought and any assumptions and simplifications made. | pg 5; line #102-105 |
| Critical appraisal of individual sources of evidence | 12 | If done, provide a rationale for conducting a critical appraisal of included sources of evidence; describe the methods used and how this information was used in any data synthesis (if appropriate). | N/A |
| Synthesis of results | 13 | Describe the methods of handling and summarizing the data that were charted. | pg 5; line #101-115 |
| **RESULTS** | | | |
| Selection of sources of evidence | 14 | Give numbers of sources of evidence screened, assessed for eligibility, and included in the review, with reasons for exclusions at each stage, ideally using a flow diagram. | pg 5; line #118-121, figure #1 |
| Characteristics of sources of evidence | 15 | For each source of evidence, present characteristics for which data were charted and provide the citations. | pg 5; line #124-137 |
| Critical appraisal within sources of evidence | 16 | If done, present data on critical appraisal of included sources of evidence (see item 12). | N/A |
| Results of individual sources of evidence | 17 | For each included source of evidence, present the relevant data that were charted that relate to the review questions and objectives. | Supplementary material table 3 and 4 |
| Synthesis of results | 18 | Summarize and/or present the charting results as they relate to the review questions and objectives. | pg 6; line #139-pg 16; line #381 |
| **DISCUSSION** | | | |
| Summary of evidence | 19 | Summarize the main results (including an overview of concepts, themes, and types of evidence available), link to the review questions and objectives, and consider the relevance to key groups. | pg 16; line #384-442 |
| Limitations | 20 | Discuss the limitations of the scoping review process. | pg 18; line #445-449 |
| Conclusions | 21 | Provide a general interpretation of the results with respect to the review questions and objectives, as well as potential implications and/or next steps. | pg 19; line #452-473 |
| **FUNDING** | | | |
| Funding | 22 | Describe sources of funding for the included sources of evidence, as well as sources of funding for the scoping review. Describe the role of the funders of the scoping review. | Title document pg #1 |

**Table 2: Full electronic search strategy for MEDLINE and CINAHL database.**

| **Search #** | **Search terms** | **Limiters/Expanders** | **Last Run Via** | **Results** |
| --- | --- | --- | --- | --- |
| **Medline Database** | | | | |
| S1 | ( ( (MM "Sex Offenses+") OR (MH "Violence+") OR (MH "Sexual Trauma") OR (MM "Sexual Harassment") OR "sexual violence" OR (MH "Gender-Based Violence") OR "sexual abuse" OR "sexual assault" OR rape ) ) AND ( ((MM "Universities") OR (MM "Students") OR "university students" OR (MH "Students, Dental") OR (MH "Students, Public Health") OR (MH "Students, Health Occupations") OR (students in higher education) OR undergraduate OR campus) ) | Expanders - Apply related words; Apply equivalent subjects Search modes - Find all my search terms | Interface - EBSCOhost Research Databases Search Screen - Advanced Search Database - MEDLINE | 968 |
| S2 | ( ( (MM "Sex Offenses+") OR (MH "Violence+") OR (MH "Sexual Trauma") OR (MM "Sexual Harassment") OR "sexual violence" OR (MH "Gender-Based Violence") OR "sexual abuse" OR "sexual assault" OR rape ) ) AND ( ((MM "Universities") OR (MM "Students") OR "university students" OR (MH "Students, Dental") OR (MH "Students, Public Health") OR (MH "Students, Health Occupations") OR (students in higher education) OR undergraduate OR campus) ) | Expanders - Apply related words; Apply equivalent subjects Narrow by Language: - english Search modes - Find all my search terms | Interface - EBSCOhost Research Databases Search Screen - Advanced Search Database - MEDLINE | 966 |
| S3 | ( ( (MM "Sex Offenses+") OR (MH "Violence+") OR (MH "Sexual Trauma") OR (MM "Sexual Harassment") OR "sexual violence" OR (MH "Gender-Based Violence") OR "sexual abuse" OR "sexual assault" OR rape ) ) AND ( ((MM "Universities") OR (MM "Students") OR "university students" OR (MH "Students, Dental") OR (MH "Students, Public Health") OR (MH "Students, Health Occupations") OR (students in higher education) OR undergraduate OR campus) ) | Expanders - Apply related words; Apply equivalent subjects Narrow by SubjectAge: - adult: 19-44 years Narrow by Language: - english Search modes - Find all my search terms | Interface - EBSCOhost Research Databases Search Screen - Advanced Search Database - MEDLINE | 446 |
| S4 | ( ( (MM "Sex Offenses+") OR (MH "Violence+") OR (MH "Sexual Trauma") OR (MM "Sexual Harassment") OR "sexual violence" OR (MH "Gender-Based Violence") OR "sexual abuse" OR "sexual assault" OR rape ) ) AND ( ((MM "Universities") OR (MM "Students") OR "university students" OR (MH "Students, Dental") OR (MH "Students, Public Health") OR (MH "Students, Health Occupations") OR (students in higher education) OR undergraduate OR campus) ) | Expanders - Apply related words; Apply equivalent subjects Narrow by SubjectGeographic: - ethiopia Narrow by SubjectGeographic: - tanzania Narrow by SubjectGeographic: - africa south of the sahara Narrow by SubjectGeographic: - africa Narrow by SubjectGeographic: - nigeria Narrow by SubjectGeographic: - uganda Narrow by SubjectGeographic: - south africa Narrow by SubjectAge: - adult: 19-44 years Narrow by Language: - english Search modes - Find all my search terms | Interface - EBSCOhost Research Databases Search Screen - Advanced Search Database - MEDLINE | 27 |
| **CINAHL database** | | | | |
| S1 | ( ( (MM "Sex Offenses+") OR (MH "Violence+") OR (MH "Sexual Trauma") OR (MM "Sexual Harassment") OR "sexual violence" OR (MH "Gender-Based Violence") OR "sexual abuse" OR "sexual assault" OR rape ) ) AND ( ((MM "Universities") OR (MM "Students") OR "university students" OR (MH "Students, Dental") OR (MH "Students, Public Health") OR (MH "Students, Health Occupations") OR (students in higher education) OR undergraduate OR campus) ) | Expanders - Apply related words; Apply equivalent subjects Search modes - Find all my search terms | Interface - EBSCOhost Research Databases Search Screen - Advanced Search Database - CINAHL with Full Text | 3,374 |
| S2 | ( ( (MM "Sex Offenses+") OR (MH "Violence+") OR (MH "Sexual Trauma") OR (MM "Sexual Harassment") OR "sexual violence" OR (MH "Gender-Based Violence") OR "sexual abuse" OR "sexual assault" OR rape ) ) AND ( ((MM "Universities") OR (MM "Students") OR "university students" OR (MH "Students, Dental") OR (MH "Students, Public Health") OR (MH "Students, Health Occupations") OR (students in higher education) OR undergraduate OR campus) ) | Expanders - Apply related words; Apply equivalent subjects Narrow by Language: - english Search modes - Find all my search terms | Interface - EBSCOhost Research Databases Search Screen - Advanced Search Database - CINAHL with Full Text | 3,323 |
| S3 | ( ( (MM "Sex Offenses+") OR (MH "Violence+") OR (MH "Sexual Trauma") OR (MM "Sexual Harassment") OR "sexual violence" OR (MH "Gender-Based Violence") OR "sexual abuse" OR "sexual assault" OR rape ) ) AND ( ((MM "Universities") OR (MM "Students") OR "university students" OR (MH "Students, Dental") OR (MH "Students, Public Health") OR (MH "Students, Health Occupations") OR (students in higher education) OR undergraduate OR campus) ) | Expanders - Apply related words; Apply equivalent subjects Narrow by SubjectAge: - adult: 19-44 years Narrow by Language: - english Search modes - Find all my search terms | Interface - EBSCOhost Research Databases Search Screen - Advanced Search Database - CINAHL with Full Text | 1,256 |
| S4 | ( ( (MM "Sex Offenses+") OR (MH "Violence+") OR (MH "Sexual Trauma") OR (MM "Sexual Harassment") OR "sexual violence" OR (MH "Gender-Based Violence") OR "sexual abuse" OR "sexual assault" OR rape ) ) AND ( ((MM "Universities") OR (MM "Students") OR "university students" OR (MH "Students, Dental") OR (MH "Students, Public Health") OR (MH "Students, Health Occupations") OR (students in higher education) OR undergraduate OR campus) ) | Expanders - Apply related words; Apply equivalent subjects Narrow by SubjectGeographic: - africa Narrow by SubjectAge: - adult: 19-44 years Narrow by Language: - english Search modes - Find all my search terms | Interface - EBSCOhost Research Databases Search Screen - Advanced Search Database - CINAHL with Full Text | 13 |

**Table 3: Summary of various forms of sexual violence from selected articles.**

| **Author** | **Year** | **Country** | **Participants** | **Sample** | **Instrument** | **Type of SV measured** | **Prevalence (%)** | | |
| --- | --- | --- | --- | --- | --- | --- | --- | --- | --- |
|  |  |  |  |  |  |  | **Lifetime** | **Post admission** | **Past 12 months** |
| 1. A. A. Ogunfowokan | 2023 | Nigeria | Female and male students | 167 | Sexual Experiences Survey–Short Form Victimization Scale | Sexual Violence |  |  | 21.9 |
|  |  |  |  |  |  | Sexual Harassment |  |  | 16.1 |
|  |  |  |  |  |  | Attempted Rape |  |  | 12.3 |
|  |  |  |  |  |  | Completed Rape |  |  | 2.6 |
| 1. A. Henock | 2015 | Ethiopia | Female students | 604 | WHO Multi-Country Study on Women’s Health and Domestic Violence against Women Questionnaire | Sexual Violence | 75.4 |  |  |
|  |  |  |  |  |  | Sexual Harassment |  | 66.3 | 63.0 |
|  |  |  |  |  |  | Attempted Rape | 33.5 | 19.6 | 13.7 |
|  |  |  |  |  |  | Completed Rape | 20.7 | 13.5 | 8.9 |
| 1. A. I. Ajayi | 2021 | South Africa | Female students | 451 | Developed by authors | Sexual Violence | 37.9 |  | 25.2 |
| 1. A. Mamaru | 2015 | Ethiopia | Female students | 385 | Developed by authors | Sexual Harassment   - Physical | 78.2 |  |  |
|  |  |  |  |  |  | - Verbal | 90.4 |  |  |
|  |  |  |  |  |  | - Nonverbal | 80.0 |  |  |
| 1. B. C. Akpunne | 2020 | Nigeria | Female students | 500 | Sexual Experiences survey (SES) | Sexual Harassment | 64.2 |  |  |
|  |  |  |  |  |  | Sexual Coercion | 9 |  |  |
|  |  |  |  |  |  | Attempted Rape | 8.6 |  |  |
|  |  |  |  |  |  | Completed Rape | 7.4 |  |  |
| 1. B. Tolesa | 2015 | Ethiopia | Female students | 605 | WHO Multi-Country Study on Women’s Health and Domestic Violence against Women Questionnaire | Sexual Violence | 66 |  | 36.5 |
|  |  |  |  |  |  | Attempted Rape | 25.1 |  |  |
|  |  |  |  |  |  | Completed Rape | 10.9 |  |  |
| 1. F. Abubeker | 2021 | Ethiopia | Female students, second and third year | 302 | Developed by authors | Sexual Violence |  | 46.6 |  |
|  |  |  |  |  |  | Attempted Rape |  | 36.7 |  |
|  |  |  |  |  |  | Completed Rape |  | 28.8 |  |
| 1. G. Mutinta | 2022 | South Africa | Female students | 600 | Developed by authors | Sexual Violence | 46.7 |  |  |
|  |  |  |  |  |  | Attempted Rape | 17.2 |  |  |
|  |  |  |  |  |  | Completed Rape | 13.5 |  |  |
| 1. H. L. Esayas | 2023 | Ethiopia | Female night students, 2nd yr and above | 330 | WHO Multi-Country Study on Women’s Health and Domestic Violence against Women Questionnaire | Sexual Violence | 83.6 |  | 49.4 |
|  |  |  |  |  |  | Attempted Rape | 37.0 |  | 6.96 |
|  |  |  |  |  |  | Completed Rape | 27.9 |  | 13.9 |
| 1. H. Workye | 2023 | Ethiopia | Female students | 393 | Developed by authors | Sexual Violence | 47 | 34.8 | 28 |
| 1. I. H. Mosha | 2022 | Tanzania | Female first year students | 268 | Developed by authors | Sexual Harassment |  | 88 |  |
| 1. J. E. Umana | 2014 | Nigeria | Female students | 1355 | WHO Multi-Country Study on Women’s Health and Domestic Violence against Women Questionnaire | Intimate Partner Violence | 42.3 |  |  |
| 1. M. Birkie | 2020 | Ethiopia | Female students | 299 | Sexual Abuse History Questionnaire | Sexual Violence | 35.1 |  |  |
| 1. M. Boladale | 2015 | Nigeria | Female and male students | 400 | Conflict Tactic Scale–Revised (CTS-R) | Dating Violence |  |  | 34 |
| 1. M. G. Negero | 2019 | Ethiopia | Female students and staff | 766 | WHO Multi-Country Study on Women’s Health and Domestic Violence against Women Questionnaire | Sexual Violence | 18.9 | 8.74 | 7.44 |
|  |  |  |  |  |  | Attempted Rape |  | 10.44 | 11.48 |
|  |  |  |  |  |  | Completed Rape |  | 3.13 | 3.13 |
| 1. M. Margaret | 2014 | Nigeria | Female students residing in university hostels for atleast 1 yr | 413 | Developed by authors | Sexual Violence | 46.7 |  |  |
| 1. M. T. Machisa | 2021 | South Africa | Female students | 1293 | WHO Multi-Country Study on Women’s Health and Domestic Violence against Women Questionnaire | Intimate Partner Violence |  |  | 43 |
|  |  |  |  |  |  | Non-partner Rape |  |  | 8 |
| 1. O. U. A. Prosper | 2014 | Nigeria | Female students | 280 | Developed by authors | Completed Rape | 16.4 |  |  |
| 1. R. Fielding-Miller | 2021 | Eswatini | Female students | 372 | Sexual Experiences Survey–Short Form Version (SES-SFV) | Sexual Violence | 60.48 |  | 44.35 |
|  |  |  |  |  |  | Sexual Assault | 51.61 |  | 37.90 |
|  |  |  |  |  |  | Completed Rape | 29.30 |  | 19.62 |
| 1. R. Sarah | 2017 | Ghana | Female and male students | 1036 | Developed by authors | Sexual Coercion | M 9.9  F 21 |  |  |
| 1. S. Gebrie | 2022 | Ethiopia | Female students | 435 | Developed by authors | Sexual Violence | 39.2 |  |  |
|  |  |  |  |  |  | Sexual Harassment | 34.7 |  |  |
|  |  |  |  |  |  | Completed Rape | 18 |  |  |
|  |  |  |  |  |  | Completed Rape | 13.5 |  |  |
| 1. S. Kassa | 2019 | Ethiopia | Female students | 402 | Developed by authors | Sexual Violence |  |  | 34.1 |
|  |  |  |  |  |  | Sexual Coercion | 59.7 |  |  |
|  |  |  |  |  |  | Attempted Rape | 21.4 |  | 7 |
|  |  |  |  |  |  | Completed Rape | 13.7 |  | 1.2 |
| 1. S. Pengpid | 2016 | Cameroon | Female and male students | 16979 | Conflict Tactic Scale–Revised (CTS–Revised (CTS-R) | Sexual Violence | M 22.7  F18.8 |  |  |
|  |  | Ivory Cost |  |  |  |  | M 8.9  F26.8 |  |  |
|  |  | Madagascar |  |  |  |  | M 5.3  F 11.1 |  |  |
|  |  | Mauritius |  |  |  |  | M 5.0  F 5.3 |  |  |
|  |  | Namibia |  |  |  |  | M 15.0  F 15.0 |  |  |
|  |  | Nigeria |  |  |  |  | M 11.7  F 14.8 |  |  |
|  |  | South Africa |  |  |  |  | M 12.5  F 9.8 |  |  |
| 1. S. Yemsrach Kebede | 2017 | Ethiopia | Female students | 395 | Developed by authors | Sexual Coercion | 43.3 |  |  |
|  |  |  |  |  |  | Forced Sexual Initiation |  | 1st yr - 41.2 >2nd yr - 8.8 |  |
|  |  |  |  |  |  | Attempted Rape | 23.4 | 1st yr - 30.9 >2nd yr -11.1 |  |
|  |  |  |  |  |  | Completed Rape | 8.7 | 1st yr - 32.1 >2nd yr -10.7 |  |
| 1. T. Abulie | 2014 | Ethiopia | Female students, 2nd year and above | 397 | Developed by authors | Sexual Coercion | 41.1 |  |  |
|  |  |  |  |  |  | Attempted Rape | 5.0 |  |  |
|  |  |  |  |  |  | Completed Rape | 6.8 |  |  |
| 1. T. Bekele | 2014 | Ethiopia | Female students, 2nd year and above | 590 | WHO Multi-Country Study on Women’s Health and Domestic Violence against Women Questionnaire | Sexual Coercion | 76.4 |  |  |
|  |  |  |  |  |  | Sexual Coercion |  |  | 43.7 |
|  |  |  |  |  |  | Sexual Violence | 41.3 |  | 31.9 |
|  |  |  |  |  |  | Attempted Rape | 23.1 |  | 6 |
|  |  |  |  |  |  | Completed Rape | 20.8 |  | 6.9 |
| 1. T. Benti | 2015 | Ethiopia | Female students | 562 | Developed by authors | Sexual Violence | 41.3 |  | 31.9 |
|  |  |  |  |  |  | Attempted Rape | 23.1 |  | 6 |
|  |  |  |  |  |  | Completed Rape | 20.8 |  | 6.9 |
| 1. T. H. Oni | 2019 | South Africa | Female and male students residing in campus | 342 | Developed by authors | Sexual Harassment |  |  | M - 33.5  F - 41.4 |
|  |  |  |  |  |  | Forced Sexual Initiation |  | M - 5.1  F - 5.4 |  |
|  |  |  |  |  |  | Attempted Rape |  | M - 2.6  F - 3.8 |  |
|  |  |  |  |  |  | Completed Rape |  | M - 1.3  F - 2.7 |  |
| 1. W. Z. Temesgan | 2021 | Ethiopia | Female night students | 422 | Developed by authors | Sexual Violence | 27.1 | 21.3 | 17.2 |
| 1. Y. M. Adinew | 2017 | Ethiopia | Female students | 462 | WHO Multi-Country Study on Women’s Health and Domestic Violence against Women Questionnaire | Sexual Violence | 45.4 | 36.1 | 24.4 |
|  |  |  |  |  |  | Attempted Rape |  |  | 15.2 |
|  |  |  |  |  |  | Completed Rape | 15.3 | 8 | 2.3 |
| 1. Z. O. Odufuye | 2020 | Nigeria | Female and male students | 399 | - | Sexual coersion | 32.0 |  |  |

**Table 4:** **Summary of key findings from selected articles (prevalence data excluded).**

| **Author** | **Year** | **Country** | **Focus of the study** | **Terminology used** | **Study design, evidence type** | **Target population** | **Key findings** |
| --- | --- | --- | --- | --- | --- | --- | --- |
| 1. A. A. Ogunfowokan | 2023 | Nigeria | Prevalence Exploring intent to use a response mechanism | Sexual Violence, Sexual Coercion, Attempted Rape, Completed Rape | Mixed methods; primary data | Female and male students and staff | **Disclosure and Reporting**   - - Reporting mechanisms were not well known by students   - Passive response by management |
| 1. A. D. Aina-Pelemo | 2021 | Nigeria | Perceptions | Quid pro quo sexual harassment | Mixed methods; primary | Female and male students | **Perceptions**   - SH is prevalent - Perception on sex for marks is influenced by institution type (public vs ptivate) |
| 1. A. Eller | 2016 | Benin | Perceptions | Sexual Harassment | Qualitative; primary data | Female and male students, professors | **Risk factors**   - students who needs better grades or money - Student clothing - Power; female students felt powerless against professors - lack of proffesional ethics - moral dilemma of - engaging in sexual relationships with students   **Consequences**   - Stigma - Saying no to professor has bad consequences, reporting him is dangerous, avoid bruising professor's ego   **Perceptions**   - Girls knows what they are getting themselves into - Men sexually harass women by pressuring or threatening them - Women sexually harass men by seducing them into doing something they shouldn’t do |
| 1. A. Henock | 2015 | Ethiopia | Prevalence Risk factors | Sexual Violence, Attempted Rape, Completed Rape | Mixed methods; primary data | Female students | **Risk factors**   - Khat chewing - Smoking cigarettes - Drinking alcohol - Having drunken friend - Lack of facilities like security fence around female domitory - Availability of substances around the campus |
| 1. A. I. Ajayi | 2021 | South Africa | Prevalence Risk factors | Sexual Violence | Quantitative; primary data | Female students | **Risk factors**   - Heavy episodic drinkers of alcohol - Insufficient financial support   **Protective factors**   - students who received adequate family financial support - students who are religious |
| 1. A. Mamaru | 2015 | Ethiopia | Prevalence Risk factors | Sexual Harassment | Quantitative; primary data | Female students | **Consequences**   - Psychological distress |
| 1. A. Melak | 2021 | Ethiopia | Factors affecting academic perfomance | Sexual harassment | Quantitative; primary | Female students | **Consequences**  Poor academic perfomance |
| 1. A. T. Opekitan | 2019 | Nigeria | Perceptions | Rape | Quantitative; primary | Female and male students | **Perceptions**   - Compare to males, females had more positive views of   - victims of rape than males   - females’ social roles involving gender - poor perception on work-related social roles   **Disclosure and Reporting** |
| 1. A. Treffry-Goatley | 2018 | South Africa | Experiences and perceptions | Sexual Violence | Qualitative; primary data | Female and male students | **Risk factors**   - Men use alcohol to hunt victims   **Disclosure and Reporting**   - When women report campus sexual abuse, security often fails to act effectively |
| 1. B. Anderson | 2022 | South Africa | Experiences | Sexual Violence | Qualitative; primary data | Female students, first years, black | **Risk factors**   - Drunk female is a target - A guy paying for alcohol is expecting sex later - Dangerous spaces: dark city (area with poor lighs) and residence rooms - First years are referred as "fresh meat"   **Consequences**   - Emotional breakdown - Drop out - Poor academic perfomance - Depression - Hate |
| 1. B. C. Akpunne | 2020 | Nigeria | Risk factor | Sexual harassment | Quantitative; primary | Female students | **Risk factors**  poly substance use |
| 1. B. M. Mapayi | 2023 | Nigeria | Experiences | Sexual Harassment | Qualitative; primary | Female and male students | **Disclosure and Reporting**   - formal reporting of cases of sexual harassment was poor - Reasons for not reporting   - Avoiding stigma   - He feelings of internal self-blame, guilt, and shame   - Poor handling of previously reported cases by the school authorities   - Protection of the perpetrators   - Highly influential positions of the perpetrator’s families   - Perception of not having enough proof   - The fear of negative repercussions following reportage   - Being begged not to report   - Unsupportive staff   - Past negative experiences with law-enforcement agents |
| 1. B. Tolesa | 2015 | Ethiopia | Prevalence Risk factors | Sexual Violence, Attempted Rape, Completed Rape | Mixed methods; primary data | Female students | **Risk factors**   - History of mother beaten by partner - Having regular boyfriend |
| 1. C. Ross | 2022 | South Africa | Perceptions and Experiences | Gender Based Violence, Intimate partner violence | Qualitative; primary data | Female students, in intimate relationships within past 12 months | **Risk factors**   - room shortages on campus - university not taking report of abuse seriously   **Protective factors**  **Consequences**   - alcohol abuse - low self-esteem - suicidal behaviours - loose focus - failing in classes - get expelled - serious injury - death by suicide or murder   **Perceptions**   - Guys have rights to get sex from their partners   **Reporting**   - Reasons for female under-reporting IPV   - not to be seen as weak   - to protect their abusive partners   - victims blamed   - women are encouraged to be submissive - Why women stay in abusive relationships   - IPV is an expression of love   - Financial insecurity   - A belief that abuse may suddenly end on its own |
| 1. D. Mehra | 2014 | Uganda | Risk factors | Sexual Coercion | Quantitative; primary data | Female and male students | **Risk factors**   - Alcohol consumption |
| 1. E. W. Dumbili | 2020 | Nigeria | Perceptions of context, risk factors | Sexual Violence | Qualitative; primary data | Female and male students | **Risk factors**   - Alcohol is used as a tool for SV |
| 1. E. Yunusa | 2023 | Nigeria | Examining strategies in addressing SV in academic institution | Sexual Coercion, Sexual Harassment | Qualitative; secondary data; internet- based documented materials |  | **Risk factors**   - Exchanging grades for sex |
| 1. F. Abubeker | 2021 | Ethiopia | Prevalence Risk factors | Sexual Violence, Gender Based Violence, Attempted Rape, Completed Rape | Quantitative; primary data | Female students, second and third year | **Risk factors**   - Young ge < 19 years - Student with partner (boyfriend or husband) - Low monthly pocket money |
| 1. F. D. Boateng | 2023 | Ivory Coast | Social norms: Date rape attitude, Myth and Violence | Sexual Violence | Quantitative; primary data | Female and male students | **Perceptions**   - Students who experienced sexual victimization/harassment, tend to adhere to greater rape myths - Students who hold rape myth views tend to demonstrate bad attitudes toward date rape |
| 1. F. Y. Gbagbo | 2023 | Ghana | Experiences | Sexual Coercion | Mixed methods; primary data | Female and male students with disability | **Risk factors**   - Inability for students to voice out their concerns in the university - Power - Lack of comprehensive policy to protect students - Lack of awareness of the existence of sexual - Coercion/harassment policy - Vulnerability as persons with disability - Poor financial status - Offensive dressing by victims - Physical challenges faced by students with - Disabilities also make them too frequently - Dependent on others for support on campus   **Consequences**   - Emotional disturbance - Feelings of shame whilst on campus/class - Pre-occupation with the harassment whenever they sit to learn - Being discouraged from participating in school/class activities - Poor concentration for studies - Negatively affecting the grades - No academic effects   **Coping mechanisms**   - Staying away from the perpetrator - Asking the person to leave them alone - Avoiding being alone with the perpetrator and telling the perpetrator that they dislike the act and should be stopped - Support from friends and significant others to contain an unavoidable situation in some cases - Fight back |
| 1. G. Eagle | 2021 | South Africa | Experiences | Sexual Harassment, Community Violence | Qualitative; primary data | Female students | **Perceptions**   - Women are highly objectified and sexualized in the taxi space |
| 1. G. Mutinta | 2022 | South Africa | Prevalence | Gender Based Violence, Sexual Violence | Quantitative; primary data | Female students | **Risk factors**   - Young age - Students with monthly allowances - Students with a sexual partner - Students staying alone in rented houses off campus - Students staying in a rented house off campus with housemates who had boyfriends   **Consequences**   - Perpetrators committing rape without using a condom (27.3%) - Survivors of rape not able to sit for an examination after being raped (46.8%) - Being impregnated and ending into an abortion (14.2%) - Being worried about being raped every time they were walking alone at night (79.2%) |
| 1. H. Kebirungi | 2021 | Uganda | Risk factors | Sexual Harassment | Qualitative; primary and secondary data | Female and male students, university managers and external stakeholders | **Risk factors**   - Poverty among students - Lecturers using power and influence to harass students sexually   **Perceptions**   - Limited awareness of SH - The culture of silence among students and management |
| 1. H. L. Esayas | 2023 | Ethiopia | Prevalence Risk factors | Sexual Violence, Sexual Harassment, Attempted Rape, Completed Rape | Quantitative; primary data | Female night students, 2nd yr and above | **Risk factors**   - Having illiterate father - Drinking alcohol - Having sexual partner - Multiple sexual partners |
| 1. H. Workye | 2023 | Ethiopia | Prevalence/Magnitude Risk factors/Determinants | Sexual Violence | Quantitative; primary | Female students | **Risk factors**   - Being a 2nd-year student having a lower educational level   **Consequences**   - Unusual vaginal discharge - Swelling/injury around genitalia - Pregnancy - Abortion - Self-blame - Fear - Anxiety - Hopelessness - Suicidal thoughts - Poor achievement/ failure in school - Withdrawal from school - Rejection from family - Rejection from friends/peers - Alcohol dependency/abuse - Sexual dependency/ abuse   Having multiple sexual partners |
| 1. I. H. Mosha | 2022 | Tanzania | Prevalence Risk factors | Sexual Harassment | Quantitative; primary data | Female students, first years | **Risk factors**   - Overcrowded vehicles - Use of public transport |
| 1. I. Mafa | 2021 | Zimbabwe | Perceptions | Sexual harassment | Qualitative; primary | Female and male Peer educators Deans of students SRC members Health personnel NGOs providing SRHR services in universities | **Consequences**   - Psycho-emotional trauma - Compromised educational output - Loss of social capital - Increased susceptibility to HIV/AIDS |
| 1. I. Mafa, T. Simango | 2022 | Zimbabwe | Response | Sexual Harassment | Qualitative; primary | Female and male Deans of students SRC representatives Health personnel NGOs giving SRHR services within universities | **Disclosure and Reporting**   - Reasons for non disclosure   - Fear of failure   - Unclear reporting systems   - Laxity from authorities in dealing with the cases   - Stigma and shame that is transferred on to the victims |
| 1. J. D. Adams | 2016 | South Africa | Experiences | Sexual Harassment | Mixed methods?; primary data | Female students, final years | **Disclosure and Reporting**   - Victims seldom report incidents of sexual harassment |
| 1. J. E. Umana | 2014 | Nigeria | Prevalence Risk factors | Intimate Partner Violence | Quantitative; primary data | Female students | **Risk factors**   - Alcohol consumption - cigarettes smoking - history of interparental violence   **Protective factors**   - Postgraduate students   **Consequences**   - Cuts, punctures, bites (55.0%) - Scratches, abrasions, bruises (48.3%) - Sprains, dislocations (18.3%) - Loss of concentration (71.1%) - Loss of self-confidence (68.9%) - School absenteeism (56.0%) |
| 1. J. Matthews | 2018 | Namibia | Perceptions, norms | Rape | Quantitative; primary | Female and male Social work students | **Perceptions**   - High rates of rape myth acceptance - Positive influence of social work education in reducing rape myth acceptance |
| 1. K. D. Konlan | 2023 | Ghana | Experiences | Sexual Harassment | Quantitative; primary | Female students, 2nd and 3rd year | **Perceptions**   - 43.0% perceived that they were at risk of sexual harassment - 30% experience sexual harrassment |
| 1. K. Peace | 2015 | South Africa | Perceptions of context | Sexual Harassment | Qualitative; primary data | Female and male students and staff | **Risk factors**   - Certain dress code from women are assumed to be inviting for sex   **Perceptions**   - Ambiguity of what constitute sexual harassment and what does not, rural vs urban context - Participant perceive that female to male SH does not exist |
| 1. L. T. Sidelil | 2022 | Ethiopia | Experiences and perceptions | Sexual Harassment | Qualitative; primary |  | **Risk factors**   - Institutional actions and inactions through which universities fail to proactively prevent and effectively respond to sexual harassment |
| 1. M. Birkie | 2020 | Ethiopia | Prevalence Risk factors | Sexual and Gender Based Violence | Quantitative; primary data | Female students | **Risk factors**   - Strict parenting style - Alcohol consumption and khat chewing - Having one or more sexual partners in life |
| 1. M. Boladale | 2015 | Nigeria | Prevalence Risk factors | Dating Violence | Quantitative; primary data | Female and male students | **Risk factors**   - Neuroticism personality |
| 1. M. G. Negero | 2019 | Ethiopia | Prevalence Risk factors Consequences | Gender Based Violence, Completed Rape | Mixed methods; primary data | Female students and staff | **Risk factors**   - Growing up in a rural area - Poor academic performance - Students coming from tight family control - Witnessing parental conflicts - Students who did not freely discuss RH issues with family members - Lack of fence around female's domitory - Lack of programs/association for female students to participate - Substance use - Lack of facilities and recreational area in campus   **Consequences**   - Self-blame (18.8%) - Fear (15.6%) - Hopelessness (15.6%) - Depression (12.5%) - Suicide attempt and ideation (6.3%) - Low school performance family (6.3%) and friend (7.8%) - Neglect - Mistrust of other people (27%) - Low school performance (13.7%) - School dropout (2%) - Temporary physical harm (8.2%) - Permanent disability (1.6%) |
| 1. M. Margaret | 2014 | Nigeria | Prevalence Risk factors Consequences | Sexual Violence | Quantitative; primary data | Female students, residing in university hostels for atleast 1 yr | **Risk factors**   - Lower year of study - Previous victimization - Consensual sexual activity - Attending a party/social gathering - Alcohol - Drugs   **Consequences**   - Depression - Guilty |
| 1. M. R. Kaufman | 2019 | Ethiopia | Risk factors | Gender Based Violence | Qualitative; primary data | Female and male students and staff | **Risk factors**   - The low social status of female students - Female students perceived academic inferiority - Traditional relationship dynamics, such as the obligation for females to have sex when in relationship - Female students’ need of financial support - Substance use - Female students using male students for personal gain - Women wearing certain clothing and putting themselves in harm’s way   **Perceptions**   - IPV and coerced sex are common in students’ relationships - Cases of male teachers manipulating female students’ grades in exchange for sex were common - Peers identified as a main source of support - Campus resources, including a gender office, campus police, and mental health services were often viewed as ineffective - Male students expressed resentment for affirmative action policies and other supports for female students |
| 1. M. R. Kaufman | 2020 | Ethiopia | Experiences and perceptions | Gender Based Violence | Qualitative; primary data | Female and male students and staff | **Risk factors**   - Traditional gender norms and their link to gender-based violence on campus - wearing modern clothes make women appear sexually provocative, and male students may assume women dressing in modern attire are inviting sexual attention |
| 1. M. T. Machisa | 2022 | South Africa | Consequences | Intimate partner Violence, Non-partner rape | Quantitative; primary data | Female students | **Consequences**   - Depressive - PTSD symptoms - Suicidal thoughts |
| 1. M. T. Machisa | 2021 | South Africa | Prevalence Risk factors | Sexual Violence, Non-partner rape | Quantitative; primary data | Female students | **Risk factors**   - Food insecurity   **Consequences**   - Risky sexual behaviours - Mental ill-health symptoms |
| 1. N. E. Nafuka | 2014 | Namibia | Perceptions, norms | Rape | Quantitative; primary | Female and male students | **Perceptions**   - Male participants endorsed rape myths slightly more than female |
| 1. O. Akintayo Olamide | 2017 | Nigeria | Experiences | Date rape | Qualitative; primary data | Female students | **Consequences**   - Trauma   **Experiences**   - Coercive and manipulative means were used by perpetrators to achieve their objective |
| 1. O. O. Babatunde | 2022 | Nigeria | Risk factors | Date rape | Quantitative; primary data | Female and male students, undergraduates | **Perceptions**   - Gender influence attitude towards rape |
| 1. O. S. Okechi | 2023 | Nigeria | Norms towards sexual harassment | Sexual harassment | Quantitative; primary | Female and male students | **Perceptions**   - High acceptance of sexual harassment myth - Gender, age, source of sex education and perceived institutional sexual harassment policy influence attitude |
| 1. O. S. Olaleye | 2019 | Nigeria | Experiences | Non-Consensual Sex | Qualitative; primary data | Female and male students who experienced attempted and completed rape in a previous study | **Experiences**   - Females reported more physical disturbances while men reported more psychological - Female survivors adopted screming and biting while male survivors used deception or plea as methods to escape from rape - Help- seeking was a rare practice |
| 1. O. U. A. Prosper | 2014 | Nigeria | Prevalence Risk factors | Rape, Completed Rape | Quantitative; primary data | Female students | **Risk factors**   - Single - Living off campus - Party & night activity lovers - Student who attended single gender schools   **Knowledge**   - 25% students had no idea what rape is all about |
| 1. P. Mahabeer | 2021 | South Africa | Assessment of institutional policies | Gender Based Violence | Qualitative; primary data |  | **Experiences**   - University residence as a “vulnerable” space - Policies are in place, but the process to seek help is frustrating - Cope by avoiding the harasser |
| 1. P. O. Bello | 2020 | Nigeria | Evaluation of Institutional strategies in addressing SV | Sexual Harassment | Qualitative; primary and secondary data | Female and male students and staff | **Risk factors**   - The culture of silence / patriarchy - The desperation of student - Wearing of provocative dresses - Lack of political and institutional will to address the menace - Female students experience sexual violence asymmetric power-relations between students and lecturers - Transactional reasons |
| 1. R. A. Aborisade | 2014 | Nigeria | Barriers of reporting | Rape | Qualitative; primary data | Female students, graduates, rape victims | **Risk factors**   - Early years in the university   **Disclosure and Reporting**   - None reported the incidence - Barriers for not reporting   - Inability to identify assailant   - Dislike or distrust of police and justice system   - Cultural barriers to obtaining help   - Unaware of the need to report   - Fear of being stigmatised   - Fear of not being believed   - Fear of retaliation   - Financial dependence on perpetrator   - Does not want perpetrator to be prosecuted   - No use, harm has already being done   - Advised not to report by friends, family, significant others |
| 1. R. A. Aborisade | 2016 | Nigeria | Norms towards rape | Rape | Mixed methods; primary | Female and male students | **Perceptions**   - High rape myth acceptance |
| 1. R. Bashonga | 2017 | South Africa | Responses | Sexual Violence | Qualitative; Secondary | Female and male Public | **Perceptions**   - dissatisfaction with university policies on sexual assault, which are perceived as outdated and ineffective - rape culture at universities is viewed as a symptom of broader patriarchy in society |
| 1. R. Fielding-Miller | 2021 | Eswatini | Prevalence Experiences | Sexual Assault, Sexual Harassment, Completed Rape | Mixed methods; primary data | Female students | **Disclosure and Reporting**   - Not all women who experience rape labeled their experience as such - Reasons for non- disclosure   - Did not think what had happened was abuse   - Did not know who to tell   - Victim blaming attitudes and stigma   - Fear of disclosure   - Disclosing within formal institutions was likely to be simultaneously re-traumatizing and unhelpful |
| 1. R. Kanyemba | 2022 | Zimbabwe | Perceptions, Experiences | Sexual Harassment | Qualitative; primary | Female students | **Perceptions**   - Females in higher education settings are often subjects of gender ideology and stereotyping where female submission is emphasised - Sexist language use is related to a particular kind of hegemonic masculinity that condones verbal violence against female students   **Coping strategies**   - Avoidance - Withdrawal - Silence |
| 1. R. Kanyemba | 2019 | Zimbabwe | Perceptions | Sexual Harassment | Qualitative; primary | Female students | **Perceptions and Experiences**   - Normalization of verbal harassment contributes to muting victimized women, thus perpetuating a culture in which violence against women becomes part of the social milieu. |
| 1. R. Sarah | 2017 | Ghana | Prevalence Risk factors | Sexual Violence, Sexual Coercion | Quantitative; primary data | Female and male students | **Risk factors**   - Age > 22 years - First partner > 4 years older - Ever had boy/girlfriend - Female gender - Having participated in an abortion - Having engaged in transactional sex |
| 1. S. D. Compton | 2023 | Ghana | Norms on relationships and consent | Gender Based Violence | Mixed methods; primary data | Female and male students | **Risk factors**   - Traditional gender roles - man is authoritative, head, women should depend on men financially and be submissive - Man decides when to have sex - Sex should be between married couple - Men are unable to control themselves once they are aroused - Men have high libido   **Perceptions**   - Relationship based consent; marriage vs dating (violence is more acceptable in marriage) - consent is given when starting a relationship - woman raped by boyfriend wont be believed - Cheating warrants violence, this is acceptable - Consent is ambigious, given in non verbal communication rather than verbal |
| 1. S. D. Rominski | 2017 | Ghana | Norms towards rape | Rape | Quantitative; primary data | Female and male students | **Perceptions**   - High level of rape myth acceptance |
| 1. S. Gebrie | 2022 | Ethiopia | Prevalence Risk factors Experiences Norms, attitudes, perceptions | Sexual Violence, Gender Based Violence, Attempted Rape, Completed Rape | Mixed methods; primary data | Female students | **Risk factors**   - Older age >24 years - Had tight family control - Had witnessed father abuse mother at childhood - Had a drunken female or boyfriend   **Protective factors**   - Female college students who have a family discussion on reproductive health and related   **Consequences**   - Injury around the genitalia (63.6%) - Swelling around the genitalia (42.2%) - Fear and anxiety (81.1%) - Poor academic achievement (67.9%) - Rejection from friends 28 (52.8%) |
| 1. S. Gukurume | 2023 | Zimbabwe | Experiences, Responses | Sexual Violence | Qualitative; primary | Female and male students, staff | **Risk factors**   - Substance abuse - Patriarchy and toxic masculinities |
| 1. S. Kassa | 2019 | Ethiopia | Prevalence Risk factors | Sexual Coercion, Sexual Violence, Attempted Rape, Completed Rape | Quantitative; primary data | Female students | **Risk factors**   - Have sexual intercourse - Have seen pornographic image - Witness history of their mother beaten by partner during their childhood - Family getting separated   **Protective factors**   - Stable financial support - Having good knowledge about sexual coercion |
| 1. S. M. H. Sadati | 2021 | Ethiopia | Perceptions of context, strategies to address | Sexual and Gender Based Violence | Qualitative; primary data | Female and male instructors | **Risk factors**   - Toxic masculinity culture - Lack of knowledge and awareness - Economic conditions - Lack of rules and regulations - Religious beliefs - Young age and less experience - Differences in individual attitudes and understanding from managers, instructors, staff members and students - Poor physical infrastructure - shortage of toilets and facilities for females   **Consequences**   - Unwanted pregnancy and its consequences - Effects on females’ academic achievements - Effects on females’ career and economic statusHealth - problems for females - Unbalanced development of the country |
| 1. S. Manik | 2021 | South Africa | Experiences, Risk factors | Gender Based Violence | Qualitative; primary data | Female students, second and third years | **Risk factors**   - Seraching for accomodation - Men showing up their power - Students who lack funding - Being female |
| 1. S. O. Okafor | 2022 | Nigeria | Experiences and perceptions | Sexual Harassment | Quantitative; primary | Female students | **Willingness to fight/react depends on**   - perceived objectivity of the institution in handling sexual harassment involving male staff and female students - secondary school gender (this is whether the high school they attended was girls-only secondary school or boys and girls studying in the same secondary school) - source of sex education - availability of place to report sexual harassment in the institution - religion affiliation of the students |
| 1. S. Pengpid | 2016 | Cameroon, Ivory Cost, Madagascar, Mauritius, Namibia, Nigeria, South Africa | Prevalence Risk factors | Sexual Violence, Intimate partner violence | Quantitative; primary data | Female and male students | **Risk factors**   - Males   - in the second and fourth year of study   - residing away from parents or guardians on their own or on campus   - coming from a wealthier family background   - made someone pregnant   - having PTSD symptoms - Females   - in the fourth year of study   - having been pregnant   - alcohol use in the context of sex   - having depressive and PTSD symptoms - Both males and females   - history of childhood physical and sexual abuse   - having had two or more sexual partners in the past 12 months   - current tobacco use |
| 1. S. Singh | 2015 | South Africa | Perception of fear | Sexual Violence, Sexual Assault | Qualitative; primary data | Female students | **Consequences**   - Fear of outsiders and male students - Fear of places like bathrooms, grounds, leisure and recreational spaces - Fear of academic spaces |
| 1. S. Singh | 2023 | South Africa | Perceptions of context, risk factors | Gender Based Violence | Qualitative; primary data | Male students | **Risk factors**   - Men’s investment in hegemonic masculinity - Conservative norms of heterosexuality - Female drinkers were targets   **Perception**   - Men’s abuse of power when intoxicated is seen as normal |
| 1. S. Yemsrach Kebede | 2017 | Ethiopia | Prevalence Risk factors Experiences, Norms | Sexual Coercion | Quantitative; primary data | Female students | **Risk factors**   - Social science student - Fathers low educational status - Mothers low educational status - Student source of support being parents - Drinking alcohol |
| 1. T. A. Olaigbe | 2021 | Nigeria | Review of contextual issues, strategies to address | Sexual Harassment | Qualitative; secondary data relevant sources of information including journals publications, textbooks, print and electronic media, reports and internet sources |  | **Risk factors**   - Factors leading to SH - LackofAcademicIntegrity - "Indecent dressing" - Declining academic standards - Inadequate security - Gender stereotype - Lack of specific policies on sexual harassment - Patriarchal configuration of the society   **Consequences**   - Psychological Problems - Lower academic standard of tertiary institutions - Loss of academic interest and school drop-out - Health challenges - Family stereotype and violation of human right   **Perception**   - Lack of universal definition of SV |
| 1. T. Abulie | 2014 | Ethiopia | Prevalence Risk factors Consequences | Sexual Violence, Sexual Coercion, Attempted Rape, Completed Rape | Quantitative; primary data | Female students, 2nd year and above | **Risk factors**   - Age of between 17 and 19 years at first sex - Use of alcohol sometimes   **Consequences**   - became pregnant - faced abortion - faced genital trauma - unusual vaginal discharge genital swelling   **Disclosure and Reporting**   - Reported (6.3%) - Reasons for not disclosing   - afraid of family, community and perpetrator   - not knowing what to do |
| 1. T. Bekele | 2014 | Ethiopia | Prevalence Risk factors | Sexual Coercion | Quantitative; primary data | Female students, 2nd year and above | **Risk factors**   - Alcoholic consumption - Current parental living condition - Childhood witnesses of maternal coercion   **Protective factors**   - Rural origin of residence |
| 1. T. Benti | 2015 | Ethiopia | Prevalence Risk factors | Sexual Coercion, Attempted Rape, Completed Rape | Quantitative; primary data | Female students | **Risk factors**   - Fear of being hit (after being battered) - Promise given to give money - Promise given to pass exam by their teachers - After they were drunk - After taking substances like chat and shisha   **Disclosure and Reporting**   - 52.1% told on one - 5.26% reported to the legal body - Reasons for no reporting   - Afraid of their parents   - Afraid of public reaction   - Afraid of the perpetuator   - Did not know that legal body is useful in such issues |
| 1. T. de Villiers | 2021 | South Africa | Adaptation of an Intervention | Sexual Violence | Qualitative; primary data | Male students, undergraduates | **Risk factors**   - Social norms impact on male behaviours, particularly male sexual behaviour - Being a successful man |
| 1. T. H. Oni | 2019 | South Africa | Prevalence Risk factors | Sexual Harassment, Attempted Rape, Completed Rape | Quantitative; primary data | Female and male students residing in campus | **Risk factors**   - Female gender |
| 1. T. S. Bezabeh | 2016 | Ethiopia | Evaluation of Institutional policies, coping mechanism | Sexual Harassment | Qualitative; primary data | Female students | - Coping strategies - staying silent - normalization of the incident - withdrawing from the academic institution - sitting for re-exams - confront the perpetrators - transferring to other departments - accepting the sexual proposition of the perpetrators - normalization  Very few female students managed to bring their sexual harassment cases either to the gender office and/or the police  Reasons of under-reporting - fear of revenge - perceived of making false allegations - lack of confidence in the institutional structures - the absence of concrete evidence - lack of awareness |
| 1. V. N. Effiom | 2019 | Nigeria | Risk factors | Sexual Harassment | Quantitative; primary data | Female and male students | **Consequences**   - Sexual harassment significantly influence student's poor attainment of grades. |
| 1. W. Z. Temesgan | 2021 | Ethiopia | Prevalence Risk factors | Sexual Violence | Quantitative; primary data | Female night students | **Risk factors**   - Residence being urban - More than 20 min taking to reach at the college - Parents living condition separately - Witnessed inter partner violence as a childhood - Alcohol intake sometimes - Having multiple sexual partner   **Consequences**   - Poor academic perfomance/ attainment of grades |
| 1. Y. L. Olaleye | 2016 | Nigeria | Knowledge gap, Evaluation of intervention | Dating Violence | Quantitative; primary data | Female and male students |  |
| 1. Y. M. Adinew | 2017 | Ethiopia | Prevalence Risk factors | Sexual Violence, Attempted Rape, Completed Rape | Quantitative; primary data | Female students | **Risk factors**   - Young age category of 20–24 - Being from rural area - Witnessing inter-parental violence as a child - Having regular boyfriend - Alcohol consumption - Having friends who drink on regular basis   **Perceptions**   - 21.9% believed it is not preventable - 19% didn’t know it is a crime   **Disclosure and Reporting**   - 19.7% - disclosed to family - 8.4% - disclosed to police |
| 1. Y. Sikweyiya | 2023 | South Africa | Risk factors | Sexual Violence | Qualitative; primary data | Male students | **Risk factors**   - Men's feelings of inadequacy - Attempts to get a female student’s attention - Sex demanded for academic favours - Sex demanded for assistance given with accommodation or financial challenges - Alcohol use   **Perceptions**   - Many tolerated/normalized acts of SV - Stranger/Non partner rape perceived as not normal and opportunistic |
| 1. Z. O. Odufuye | 2020 | Nigeria | Prevalence Risk factors Experiences;Norms, attitudes, perceptions | Sexual Coercion | Mixed methods; primary data | Female and male students | **Risk factors**   - Female gender   **Disclosure and Reporting**   - Only few sought help |

1. Shamseer L, Moher D, Clarke M, Ghersi D, Liberati A, Petticrew M, Shekelle P, Stewart LA. Preferred reporting items for systematic review and meta-analysis protocols (PRISMA-P) 2015: elaboration and explanation. Bmj. 2015 Jan 2;349. [↑](#footnote-ref-1)
